# Exploring Parent Support Needs during the Newborn Hearing Diagnosis Pathway

**DOI:** 10.3390/jcm11051389

**Published:** 2022-03-03

**Authors:** Kayla Elliott, Danya F. Vears, Valerie Sung, Zeffie Poulakis, Jane Sheehan

**Affiliations:** 1 Prevention Innovation Research Group, Murdoch Children’s Research Institute, Parkville 3052, Australia; kayla.elliott@mcri.edu.au (K.E.); valerie.sung@rch.org.au (V.S.); zeffie.poulakis@rch.org.au (Z.P.); 2 Department of Paediatrics, University of Melbourne, Parkville 3052, Australia; 3 Biomedical Ethics Research Group, Murdoch Children’s Research Institute, Parkville 3052, Australia; danya.vears@mcri.edu.au; 4 Melbourne Law School, University of Melbourne, Carlton 3052, Australia; 5 Centre for Biomedical Ethics and Law, KU Leuven, 3000 Leuven, Belgium; 6 Centre for Community Child Health, The Royal Children’s Hospital, Parkville 3052, Australia

**Keywords:** universal newborn hearing screening, hearing loss, parent perspective, support needs

## Abstract

Universal newborn hearing screening (UNHS) facilitates early detection of permanent congenital hearing loss in newborns. In recognition of specific needs among parents, support services have been established within some UNHS programs, including the Victorian Infant Hearing Screening Program (VIHSP). Despite this, there is limited research about how to best support parents in the context of well-established UNHS programs. This project aims to retrospectively explore parental support needs between the newborn hearing screen and enrolment into early intervention services. We used semi-structured interviews with parents three- to- six-months post confirmation of their newborn’s diagnosis of bilateral moderate-profound sensorineural hearing loss. Data were analysed using inductive content analysis. Thirteen parents of ten children were interviewed. Parents described high satisfaction with the support they received. Some parents felt unprepared for a diagnosis of hearing loss, having been reassured that transient causes such as middle ear fluid caused the hearing screen result. Parents reported mixed responses regarding the value of parent-mentor support along the pathway and some parents described needing additional psychological input to adjust to their child’s diagnosis. These findings provide insights into how a well-established UNHS program, VIHSP, supports parents along the hearing diagnosis pathway and how support can be further enriched.

## 1. Introduction

Children born with hearing loss are at increased risk of behavioural problems, lower academic achievement, challenges with both verbal and non-verbal communication, and poorer employment prospects [1,2]. However, early intervention and treatment of hearing loss improves overall outcomes for children with hearing loss, especially when hearing amplification and language intervention occurs in the early months of life during the critical period for language acquisition [3,4,5]. It is therefore crucial that infants born with hearing loss are identified early so there is minimal disruption to their development. Universal newborn hearing screening (UNHS) programs are an important public health service to detect congenital hearing loss in all newborns, regardless of the presence of risk factors for hearing loss [6]. The prevalence of moderate to profound bilateral congenital hearing loss is 1.3 per 1000 infants [7].

Supporting parents from hearing screening to diagnosis, and into early intervention, is a critical component of the diagnostic experience to foster the best possible long-term outcomes for the child [2,8,9]. Parents describe the pathway to their child’s hearing diagnosis as an anxious time [9]. Russ et al. concluded that there is a need for support systems to be in place when an infant is referred to diagnostic audiology as it is a time of great emotional distress and uncertainty for the parents [9]. Additionally, the delivery of their child’s hearing loss diagnosis leads to strong emotions of shock, guilt, and denial [10]. Therefore, it could be postulated that parents will have specific support needs due to the challenging emotions associated with their newborn’s hearing diagnosis. Research has shown accommodating parent support needs could aid in the effectiveness of early intervention programs for their child, as parents feel more empowered to assist with hearing habilitation [11]. In addition to screening for hearing loss, there are proposed benefits of UNHS programs embedding support services within their programs to assist parents of infants who do not pass the newborn hearing screen and require further audiological investigation. Despite the recommended benefits of pairing UNHS and support services together [1], it remains unclear how many countries have embedded support services into their screening programs.

The United States Preventative Services Task Force for Hearing Loss recommends family-centred care in the paediatric setting, stating “early intervention services for hearing-impaired infants should be designed to meet the individualised needs of the infant and family” [1] (p. 145). Family-centred care involves tailoring the delivery of health services based on parental participation and collaboration with all health professionals who provide healthcare to their child [12]. For children with hearing loss, 10 key principles have been proposed by Moeller and colleagues [13] for the implementation of family-centred care as outlined in Box 1. It is thought that, by implementing these principles to the early detection and intervention of childhood hearing loss, parents will feel more supported during their adjustment to their child’s diagnosis, so that they can then work with services to deliver the best possible healthcare for their child.

Box 1 **10 Key Principles of Family-Centred Care for Children with Hearing Loss as defined by Moeller et al. [13].**

1. Early, timely, equitable access to services
2. Family–provider relationships
3. Informed choice and decision making
4. Family social and emotional support
5. Family–infant interactions
6. Use of assistive technologies and supporting means of communication
7. Qualified providers
8. Collaborative teamwork
9. Progress monitoring
10. Programme monitoring

Given that parents may express strong feelings of shock and helplessness upon receiving their newborn’s hearing loss diagnosis [10], family-centred care enables health professionals to care for both the child and the parents by providing the necessary support required [9]. A holistic approach to care in the hearing loss setting improves the overall health outcomes of the child, fostering strong interpersonal relationships between the child and parent [13]. Additionally, higher staff satisfaction and more effective use of healthcare resources have also been associated with family-centred care [12,14]. Supporting parents’ needs is an important component of achieving the best possible outcomes of a child diagnosed with hearing loss.

In the state of Victoria, Australia, the Victorian Infant Hearing Screening Program (VIHSP) finalised the roll-out of their UNHS program in 2012 and screens the hearing of >99.5% of newborns within the first few weeks of life [15]. Approximately 0.8% of infants who have their hearing screened by the VIHSP will obtain a ‘positive screen result’, requiring further diagnostic audiological testing [16]. Approximately 40% of the newborns who are referred to diagnostic audiology will be diagnosed with a hearing loss of varying type and degree [16]. The VIHSP has an Early Support Service (ESS) embedded within the program to support families whose newborns do not pass the hearing screen. The roles of the VIHSP ESS team are to provide support and information, guide parents through the hearing diagnosis pathway, and be a contact for parents when they have any concerns or questions. Following diagnostic assessment, the ESS provides services to all newborns diagnosed with the VIHSP target condition, bilateral moderate to profound permanent hearing loss; newborns with other degrees and laterality of hearing loss are provided with support on a case-by-case basis based on available resources. While the hearing screen and ESS are both embedded within VIHSP, other services along the hearing diagnostic pathway, including diagnostic audiology, hearing amplification, early intervention services, parent groups and mentors with lived experience, and medical services are governed externally. Cochlear implantation may also be offered for newborns with severe to profound hearing loss to enable hearing for speech development. Families can seek these supports themselves or they may be offered, or recommended, by other services along the pathway. All children who are diagnosed with bilateral moderate–profound hearing loss in Australia can access hearing amplification devices and services without cost, and qualify to access the National Disability Insurance Scheme (NDIS), which provides funding to families to access early intervention services [17]. Families have the choice of two main providers for early intervention specific for hearing loss, offering oral (English) and Australian sign (Auslan) language therapies; they can also choose to access any speech therapist or Teacher of the Deaf registered as an NDIS provider.

To date, published studies exploring parent needs have been limited by recall bias, with most studies relying on parental report of their experiences a substantial time after hearing diagnosis, sometimes up to six years [18]. There are no studies exploring parental experiences within the first few months’ post-diagnosis, when parents can more accurately recall their perception of their support needs through the hearing diagnosis pathway [19]. There is limited literature exploring the barriers and facilitators to engagement with support services, the best methods for information delivery, and the optimal timing of support, including when support could be reduced. Moreover, published research to date has not examined how parents utilise internet resources. The experiences and support needs of regional families are also underrepresented in the literature. Overall, there are few data specifically exploring the parent perspective of support needs within the context of well-established UNHS programs, with most existing studies addressing parental support needs in populations where UNHS was not well-established. Since the state-wide roll-out of the VIHSP in 2012, there has been no research published in Victoria about parents’ perspectives of their support needs from the newborn hearing screen to hearing loss diagnosis. Russ et al. [9] (p. 358) suggests that UNHS programs undertake qualitative research to support “future programme planning and development <which> will enable providers to better meet the needs of children with hearing loss and to adequately support and inform parents”. This study aims to understand the parent experiences and perspectives of their support needs along the newborn hearing loss diagnosis pathway through Victoria’s UNHS program, to ultimately provide practical recommendations for optimizing service delivery through VIHSP as well as other UNHS programs in Australia and internationally.

## 2. Materials and Methods

This study involved a qualitative design using semi-structured interviews with parents to explore parental perspectives of their support needs along the newborn hearing loss diagnosis pathway. Potentially eligible families were identified from auditing the VIHSP clinical database. Families were eligible to be invited into the study if their newborn (i) had a VIHSP ‘refer’ result (positive hearing screen result requiring further diagnostic audiological confirmation), (ii) had a diagnosis of bilateral moderate–profound sensorineural hearing loss (>40 dB) confirmed between 91 and 182 days from the date of the database audit search, (iii) was the parent’s first child to complete the VIHSP hearing diagnosis pathway, and (iv) had a parent with sufficient English to participate.

Recruitment took place from May 2021 to September 2021; this occurred during a state-wide COVID-19 lockdown. The VIHSP sent all eligible families a project information letter describing the study and offering the opportunity to opt out of being contacted by the project team. This invitation process was used to ensure the project’s sample was not biased towards families who were ‘doing better’ and more likely to have the capacity to opt in. After the 14 day opt-out period, the VIHSP provided the project team with the contact details of the parents who did not opt out. The project team contacted the primary caregiver via telephone to discuss the project in detail, confirm eligibility, and, if appropriate, invite the parent to participate. During the COVID-19 pandemic, most of the support provided by ESS moved to telehealth with limited face-to-face contact due to the ongoing public health restrictions. The parents interviewed experienced varying degrees of COVID-19 public health restrictions, and as such, a limited number of families had access to home visits.

A semi-structured interview guide was used to conduct the interviews. It included questions relevant to the hearing diagnosis pathway: (1) engagement with support services, (2) parental support needs post-diagnosis, (3) timing of support services, (4) style of information delivery, and (5) opportunity to identify other issues. Participant characteristics such as age, languages spoken at home, and post code were also collected. Two pilot interviews were conducted prior to recruitment: one with a staff member from VIHSP and another with an ineligible parent with two children with profound hearing loss. The results of the pilot interviews were used to further refine the interview guide prior to conducting interviews with study participants.

Parents were offered the option to do the interviews via telephone or Zoom video call due to COVID-19 restrictions. All interviews were conducted by KE. They were audio-recorded, transcribed, de-identified, and allocated pseudonyms to protect the privacy of participants. Transcripts were analysed using inductive content analysis. This method was chosen as it is an effective way to analyse data in small, non-complex research because it aims to build up an understanding of the transcripts’ content [20]. The inductive nature of the analysis enabled the project to explore the research question and also allowed additional needs and nuances of the parent experience to be captured. This technique is useful when attempting to understand and describe a specific experience [21,22]. Segments of the text were initially coded into broad content categories and then coded into finer subcategories. KE coded all transcripts, and two other researchers, DFV and VS, coded two transcripts each to ensure rigour. Minor discrepancies between coders were resolved via discussion.

## 3. Results

### 3.1. Participant Characteristics

Within the four-month recruitment period, 23 families were identified as potentially eligible and were sent an opt-out letter. Two (9%) families opted out of contact by the study team. Of the 21 families contacted, ten families were interviewed, including thirteen parents of ten children (48%), among which were ten mothers and three fathers. The sample included two families from regional Victoria (15%). The remaining 11 families either declined to participate or did not respond to any of the recruitment attempts by the study team. Interviews were conducted via telephone or Zoom video calls. Interviews ranged in duration from 20 to 54 min. Table 1 summarises the participant characteristics.

### 3.2. Parental Support Needs following the Newborn Hearing Screen

The exploration of parental experience of the hearing diagnosis pathway for newborns revealed seven major content categories. These included (1) support needs pre-diagnosis, (2) parent experience of the information received following the newborn hearing screen, (3) experience of audiology appointments, (4) support needs post-diagnosis, (5) timing of support services, (6) method of information delivery, and (7) the impact of COVID-19 on the support provided. Illustrative quotes are used to represent the data.

#### 3.2.1. Parents’ Support Needs Pre-Diagnosis

Most parents felt supported prior to diagnosis and were comforted knowing they had access to a support worker to whom they could ask questions. Parents reported that receiving reassurance, knowledge about hearing services, and information about next steps were the most important forms of support.

“I think just explaining what the next steps would be, in terms of what testing he would have to get done, and where that would be. Sort of the time frames of things as well. So just sort of giving us a real clear picture of the path that we were going to take, in terms of giving my child the support and intervention that he needed”

—Cat

One family expressed that they would have liked more information about hearing loss earlier on.

“In between the audiology test and the screening there were only a few pamphlets that we got that were useful. But I think that we had, it was only because he was diagnosed with hearing loss, I think if we had more information about hearing loss it would have made us feel a little bit better.”

—Ivy

Some parents struggled to identify support needs before the confirmed diagnosis but still appreciated the contact from the support service.

“I don’t think we really needed much during that period. We sort of just thought, like he’s not deaf, we will just go to another appointment and sort it out. So, it was nice to have ESS call us and kind of have that bridging.”

—Georgia

The pathway to diagnosis was described by parents as busy, and often families forgot that there was a support service they could access. Parents were therefore grateful that the support service proactively reached out to them because they found the support valuable.

“I really appreciated the fact that I didn’t have to reach out for <the support> because when we first got that diagnosis your world just becomes what? You are suddenly introduced to a whole new world, and you just don’t know, you don’t know what you don’t know yet, so the fact that all these supports were the ones calling us <…>, they were the ones that reached out, it made such a difference for us”

—Marnie

When parents were asked about whether they required any additional support in the early days, most expressed that they did not. However, some parents spoke of the lengthy time it took to obtain a confirmed diagnosis as a source of frustration. One family suggested support workers should try to contact both parents.

“I just feel, it could be more fast. Yes, like instead of we waited for three months to get all these things done, like getting him hearing aids, it could be more early like around one month when he was only a couple of weeks old.”

—Hazel

“I made comment about that to my wife and said it’s sort of fine that they ask about how I’m going, and they ask how I feel about things but I actually wasn’t contacted. She then ended up speaking to <support worker> about it I believe and then <support worker> contacted me and was good from then on, in terms of, she did contact me a bit. But I found it interesting that the child has two parents and they managed to get at least, whether they had my phone number or not, I didn’t get the call initially.”

—Kaiden

#### 3.2.2. Parents’ Experiences of Information Received following the Newborn Hearing Screen

Many parents felt they were overly reassured that the positive screen result occurred due to transient causes, such as fluid in the ears. A few parents commented that receiving this information set up an unrealistic expectation that there was nothing to worry about and were therefore unprepared for the eventual hearing loss diagnosis.

“When we were getting the newborn hearing test in hospital, we kept getting told over and over and over it’s probably just fluid from everyone, so nurses, midwives, the person doing the test, the paediatricians <…> and I understand for a lot of kids it is probably just fluid but the fact that we weren’t, <…> nobody said but if she doesn’t <have fluid> this is what it is going to be like, it is not as terrifying as you think it is. So that was something we kind of found very difficult to sort of be okay with, we weren’t prepared for it if it was not fluid”

—Marnie

Multiple parents suggested how they would like information delivered after the positive screen result. Some reflected that the diagnosis might not have been as much of a shock if hearing screeners explained the possibility of a hearing loss diagnosis.

“You need a bit more, realistic facts, they did tell us some percentages and stuff, but just say because he has failed, he could very well be deaf, and you need to go and get the test done. Not like don’t worry about it too much, just go get it done, kind of fuzz (gloss) over it. Maybe because it’s easier for them to not have to worry about the intensity of all the emotions and questions and stuff I don’t know”

—Georgia

In contrast, one parent thought being told extra information at the time of the hearing screen would have been overwhelming.

“I remember asking the lady, does this happen. And she said it does, with some children. But <the hearing screener> was probably being a bit kind in the sense that in hindsight, I can imagine that she would have been seeing it as oh my daughter has failed it, this probably isn’t a good thing, but she was also talking to a new mum in hospital on her own, it was COVID lockdown. So, I’m sure she was probably thinking this isn’t good, but <hearing screener> wasn’t telling me that and I probably would have preferred her not to anyway.”

—Daisy

#### 3.2.3. Parent Experience of Diagnostic Audiology Appointments

Many parents found information provided from the audiologist to be valuable, although parents found the duration and number of audiology appointments challenging, with some parents experiencing difficulties with audiologists. One parent felt they left the audiology appointment empty handed and would have liked some written resources to help them process the diagnosis.

“I do not think going to those appointments were easy, they were a bit annoying, they were a bit of a drain those audiology appointments to be honest because there were so many”

—Annie

“The audiologist she was awesome, she was really compassionate, and she answered any questions, she took her time with us, but maybe just, you feel like you leave empty handed. You’re told your child is deaf and then that’s it. You’re told okay you’ll get a phone call and we will start booking in appointments and I remember her saying that there is going to be lots of appointments coming up so we don’t want to overload you with information now.”

—Georgia

#### 3.2.4. Parents’ Support Needs Post-Diagnosis

A variety of support needs post-diagnosis were described by parents, such as needing to understand audiology results and the type of hearing loss their child has. Additionally, guidance on next steps and referrals to early intervention services were highly valued by all parents. Most parents felt reassured once the support worker informed them about the services they could access for a child with hearing loss.

“We did have a lot of things that we did worry about mainly around his learning and if he would develop any kind of learning impairments or speech impairments or anything like that and how it may restrict him growing up or him being able to do the tasks that someone who didn’t have any kind of impairments would be able to do. But then once we kind of had a chance to process a bit more, we’ve come along okay, we have realised it’s more common than we think. And then with the support kind of helped us gain a better understanding of it, helped us learn how to kind of deal with it and how to approach it as well because we just didn’t really know what to think of it when we first found out”

—Jackson

Overwhelmingly, parents reported that the most important form of support post-diagnosis was being enrolled into early intervention programs.

“<support worker> was the one who linked us with these organisations, <agency names>. Before talking to them, she actually explained the background of both agencies and yes, so from her, I came to know what kind of service both agencies provided, and which will be for good for all of us, the whole family as well.”

—Hazel

Parents identified many resources that were helpful including information for extended family members, funding, and practical tips to help a child with hearing loss. However, some parents suggested they would have liked some more information on Australian Sign Language known as Auslan.

“But I think maybe like there’s kind of not enough serious sort of exposure to Auslan and also being non-verbal and being deaf. Like it’s also okay if you want your child to be deaf and have them go to a deaf school and use Auslan, you know that’s also perfectly fine”

—Georgia

When parents were asked about additional support requirements post-diagnosis, half described wanting supplementary therapeutic support to cope with the grief associated with the diagnosis.

“I think, what really sort of surprised me was post the diagnosis we probably wanted to speak to someone more <...> just sort of in and around it and the feelings and those sorts of things, but we actually found that pretty hard too, in terms of finding someone, and someone who specialised in that area too”

—Felix

Other additional support needs reported by a few parents included more help understanding how the National Disability Insurance Scheme (NDIS) works and further support from services such as the audiologist.

“I think we needed a bit more support like from the audiologists and maybe the paediatrician, and the medical staff that were involved that have knowledge and experience and know avenues, we found that they didn’t really step up in anyway.”

—Belle

Parent mentor support, where parents connect with other families who also have a child with hearing loss, was discussed by many parents. Some parents felt they benefitted from learning from someone with lived experience.

“But you speak to families, <...> their son was the same age as my second son and he had CMV <Cytomegalovirus> as well and had hearing loss and cochlear implants and we connected with them and I think that’s a good thing as well, maybe just having families that they can get in contact with, and we still have frequent contact with them now. I think just because it’s new to you and your family, if you don’t get that support, I think you need to get it from people who have gone through or going through the same thing as you.”

—Eleanor

Others found parent mentor support overwhelming, especially when offered in the early days.

“Parents of deaf children and stuff which I think was meant to be helpful, but I actually found that really confronting at the time ... I think the fact that the other people have done this, and their stories are wonderful, and I just don’t think initially I was ready to hear about everyone else’s child.”

—Lucy

However, most families did feel reassured by speaking to people who had been through a similar experience. Some parents spoke highly of social media as a form of support, with one mother setting up an Instagram account to document her child’s hearing loss journey. The Facebook group of one particular philanthropic organisation was referenced by multiple parents as being a great way to connect with others, especially for those who were reticent to connect directly with other families.

“Actually, that’s another place, that I have felt quite supported in, Facebook Groups, like Aussie Deaf Kids. I have got a lot of reassurance from other people posting questions and then reading other people’s answers and experiences, that has been really reassuring.”

—Marnie

#### 3.2.5. Timing of Support along the Hearing Diagnosis Pathway

Although parents found the diagnostic journey to be overwhelming, all parents appreciated how quickly the support service proactively contacted them and how it was very easy to access ESS whenever they needed.

“I think it was really perfectly timed. If I recall, I think we heard from ESS <...> definitely well before the first full audiology test. And that was excellent because it meant we were sort of walking in there knowing who we were going to meet, and what the process would involve and ESS explained yeah how to best prepare for that appointment, and the timing of it was great. <...> I was really impressed with how quickly we were followed up after we had been to the appointment.”

—Cat

The majority of parents agreed that the two times when most support was needed were after they received the positive screen result and the confirmed diagnosis.

“The day before the next hearing screening test I was a little bit anxious I guess because I had never experienced that before and then yeah, he failed that one and I just burst into tears because I didn’t really know what that meant or what was going to happen”

—Eleanor

“Definitely when we first got the diagnosis. I think, just knowing what was out there, that was huge for us, we had no knowledge at all just what services were out there, what was available, what the deaf learnt, now what happens next, like we have just been told she’s deaf but what actually happens so yeah that was when we needed it the most, the support I guess, kind of right after we got the diagnosis”

—Daisy

When parents were asked whether they had enough time to process both verbal and written information from the support workers, many replied that they did. A few parents commented that they valued having autonomy over the pace of support as well.

“It was certainly overwhelming, I think the timeline, because we didn’t want to like delay anything or like slow down the process of making sure that he gets the right support that he needs, I think the timing was good, even though at the time it was a lot going on.”

—Ivy

Parents found that support from ESS naturally reduced once they were enrolled with early intervention services.

“We sort of have naturally had less questions once we have been referred to <agency names>, <...> and we commenced our appointments with Hearing Australia, and he’s got his hearing aids. I think at that point we naturally started to sort of refer to those services rather than the Early Support Service.”

—Cat

Yet one parent did feel they would have benefited from prolonged support from ESS.

“If <support worker> was still contacting us now, I feel like we would be more ready to talk about things. Obviously, you accept what’s going a lot better because it is what it is. Life has settled down a bit, the other kids know what’s going on. I think that, after that first two months, three months, would be more beneficial, with early support stuff, when I say more beneficial, you do need the contact early because you are shocked but in terms of actually getting something out of it, I think early days they get you through it and then now we would be getting something out of it.”

—Kaiden

#### 3.2.6. Method of Information Delivery

Parents were satisfied with how they received information from the support service, with most preferring verbal phone contact initially with follow up emails and physical brochures posted. Many parents also referred to Aussie Deaf Kids as a reliable and informative website.

“I liked hearing it over the phone I guess in the beginning and then yeah I found both really helpful but yeah I still think you need the phone support as well as getting it in the mail”

—Annie

“I think the best thing for me was being referred to good websites that had accurate information like Aussie Deaf Kids. <...> each day I would read like just a bit, like whether it was someone’s story or something about the services available or trying to think what else. Just all the different things.”

—Daisy

#### 3.2.7. The Impact of the COVID-19 Pandemic on the Support Service

Despite the COVID-19 Pandemic interrupting usual service delivery, parents felt they did not miss out on any information by having access to support online, rather than in-person. However, it did highlight how parents valued face-to-face support, such as home visits or attendance at audiology appointments by support workers. Additionally, a few parents also described how it was isolating receiving the diagnosis by themselves as COVID-19 density restrictions only allowed one parent to attend audiology appointments.

“I remember thinking at the time that would have been really great to have <support worker> there just to sort of like after the appointment we knew she heard what we heard and we could sort of clarify anything or debrief with her, and she would have known exactly what was discussed <...> I think that offering as part of the service is really valuable and yeah that would have been wonderful if we had that.”

—Cat (In reference to ESS support worker attending audiology appt)

One couple had a home visit in between the Melbourne lockdowns, and they highly valued it.

“I think that was really helpful as well having that face-to-face contact with someone being able to talk to someone physically in person. I think that made all the difference to our experience”

—Ivy (and partner Jackson)

Overall, the majority of parents were very impressed with the support they received and felt the support service was able to respond to their needs both pre-diagnosis and post-diagnosis.

## 4. Discussion

This study is the first to examine parental support needs within three to six months of their newborns’ confirmed hearing loss diagnosis using semi-structured interviews. This study provides insight into the perspectives of a small sample of families of newborns diagnosed with bilateral moderate to profound hearing loss, in the context of a well-established UNHS program in Victoria, and its results have implications for practice.

### 4.1. Overall Satisfaction with Support Services

The majority of parents were impressed with the support they received and appreciated having a proactive support worker. Interviews revealed that the support service was able to adapt to the individualised needs of the family by tailoring the information, guidance, and support provided, which allowed the family to be active participants in the care of their child. Ultimately, many of the support needs identified by parents were relevant to the family-centred care model as defined by Moeller et al. [13]. The family-centred care principles that closely align with the parental support needs described in this study include (1) early, timely, equitable access to services; (2) family–provider relationships; (3) informed choice and decision making; (4) family social and emotional support; and (5) progress monitoring of the family. Family-centred care and its relevance to parent support needs will be explored more below.

Parents appreciated how quickly their child was able to be scheduled into diagnostic audiology appointments, which was aided by having a support worker guide them through the different steps and prepare them for the appointment. This ties into one of the fundamental principles of family-centred care: timely, equitable access to hearing services, which include diagnosis and treatment [13]. While most thought the diagnostic process was very efficient, some families did express frustration regarding the time it took to obtain a confirmed diagnosis, which delayed their access to early intervention services. The reasons for delayed diagnosis varied due to interruptions from the pandemic, disruptions over the Christmas period, and multiple audiology appointments required for unsettled babies. DesGeorges [8] argued that parents require efficient and convenient referrals to specialists so their child can move forward in their diagnosis and intervention journey. The importance of coordinated service delivery was illustrated from a mother’s interview comment in their study: “For me, the pain is not my daughter’s diagnosis, but it is the language opportunity she lost during those 11 months between when I first asked the family doctor for help and when I cornered the ENT into finally diagnosing her” [8] (p. 91). This example demonstrates how important access to timely, quality care is to prevent unnecessary diagnosis delays, which could negatively impact a child’s development.

Parents felt that the support worker was able to assist them with making informed decisions, by providing them with detailed information about service providers and communication modalities. The overwhelming majority of parents reported being satisfied with the information they received, with many describing the information kit that ESS provided as very helpful. However, some families felt they needed more written information from the audiologist following the initial diagnosis to help them understand what hearing loss is and what was happening. This finding is consistent with Scarinci et al. [18], where caregivers were pleased with the information provided to them from services, except they too needed some extra information at the initial diagnosis; discussions with professionals were generally brief, and only limited written information was offered. Parents appreciated the delivery of information via a phone call with the support worker with follow up written materials sent either via email or post. This finding, along with the findings of others [23], supports the notion that parents find information delivered in multiple formats most helpful.

While parents did not feel that they missed out on any information as a consequence of the COVID-19 pandemic changing usual service delivery, the restrictions on face-to-face support in the form of attending audiology appointments and families’ homes highlighted how this form of support is valued by parents. A comparison of this finding with a study of children with autism spectrum disorder highlighted how both face-to-face and online sleep education were able to help parents feel supported in improving their child’s co-occurring sleep behaviour problems, suggesting that access to both face-to-face and online support is helpful for parents [24]. Home visits have been recommended by the Joint Committee on Infant Hearing Screening to achieve family-centred care and to also assist parents to engage with their child’s hearing habilitation [25]. As the pandemic continues, it is necessary to think of strategies that can mitigate risk in order to allow face-to-face support services to recommence. Conversely, strategies to maximise the effectiveness and efficiency of telehealth services is also important to ensure quality of care can continue when face to face services are unavailable.

### 4.2. The Need for Balanced Information after Positive Screen Result following the Newborn Hearing Screen

The parents’ perspective of the information received following the newborn hearing screen was a particularly important part of the parents’ experience of the hearing diagnosis pathway. Previous authors have found that parents often react in a shocked and overwhelmed way to a confirmed diagnosis [9,10,26]. However, there are no published findings regarding the need to prepare parents for the possibility of a hearing loss following a positive screen result or the best way to deliver this result to parents.

Some interviewees argued that they felt overly reassured from multiple sources that the positive screen result likely occurred due to transient causes, such as middle ear fluid, which meant that the confirmed diagnosis could have had an unexpected impact on parents. When a baby receives a positive screen result, the VIHSP screener explains the result to the family by describing three possible reasons for the positive screen result: the baby may have been unsettled during the screen, there may be a temporary blockage (e.g., fluid) in the ear, or the baby may have a hearing loss. It is possible hearing screeners may be trying to reduce anxiety for parents during a very stressful time by overly reassuring parents about the best-case scenario. At the same time, parents may have been choosing to hear one of the less distressing explanations given about a positive screen result (i.e., that it is fluid, which is temporary). While one family in this study did prefer such a reassuring approach, the overwhelming majority expressed the desire to be more informed about the possibility of hearing loss. The finding that most parents interviewed wanted more realistic facts and transparent information, yet still delivered in a caring way, is supported by research focusing on the delivery of the confirmed diagnosis by the audiologist [26]. Schmulian and Lind [26] (p. 59) found a “complex relationship between parental need for specifics and straight answers, while insisting on high levels of diplomacy and sensitivity”, which echoes what parents needed following the hearing screen in this study.

Given that 60% of babies who go to confirmatory audiology appointments will not be diagnosed with a hearing loss, hearing screeners do need to strike the right balance of preparing the parent for a hearing loss diagnosis while also not overwhelming them. Clearly, this is an area that has direct implications for practice as well as an avenue for future research. An exploratory qualitative study with a prospective design following families after a positive screen result could be conducted. This would allow the informational needs of families at each stage post screening to be explored to determine if there are any differences once the diagnostic result is known.

### 4.3. The Need for Therapeutic Support to Assist Parents to Adjust to the Diagnosis

The results from this study support the notion that parents may need further therapeutic support to deal with the adjustment and grief associated with their infant’s hearing loss diagnosis. While previous research has clearly established the emotional toll a hearing loss diagnosis has on parents [9,10,23], there appears to be a gap in the literature in terms of addressing whether a pathway to therapeutic support is necessary. Recently, Schmulian and Lind [26] interviewed ten parents whose child had hearing loss to construct an emotional lifeworld of the parent experience. They concluded that “parents require a level of emotional support that exceeds frameworks of counselling” [26] (p. 60), suggesting that basic emotional support may not be enough along the hearing diagnosis pathway. A Queensland study interviewed hearing health professionals who indicated that if a family was identified as needing more mental health support, they would refer them to separate services [27]. They found that, despite this referral pathway, one family who was interviewed in that study felt that they did not obtain the mental health support they needed.

Our study was able to build on this finding, with half of the parents interviewed describing the need for further mental health support. Family, social, and emotional support is another key pillar of family-centred care, and while ESS can provide this support initially, it appears that some parents require ongoing psychological support beyond what the ESS team can provide. This suggests that a referral pathway to psychological support could be warranted. Therefore, the first step might be to explore how psychological support has been provided to other parents whose newborns are diagnosed with permanent conditions at birth. This could then further guide investigations into the role of counselling and psychological support to aid parents whose newborn is diagnosed with hearing loss.

### 4.4. Providing Support to Both Parents

A finding that emerged from this study was the need for professionals to be cognisant of directly offering support options for both parents, with one family describing how they would have liked the support service to contact the father directly in the initial days following the hearing screen. It is possible that this finding could be reflective of the lack of face-to-face contact from support workers due to the COVID-19 restrictions, rather than standard practice. Practices were modified so that support workers contacted the primary caregiver via telephone calls, and the second/other parent was not always directly involved in the support, which may have resulted in the father being excluded. By contrast, one of the other fathers who was interviewed did obtain access to a home visit and felt included in the process. While the families interviewed in this study all had the mother as the primary caregiver, this is not always the case, particularly in families with different family structures, such as same-sex couples and single parent families. In recognition that the secondary caregiver might have different support needs, a suggestion to further enhance the service could be to provide an option for the other parent to also be contacted. Previous literature has also identified the need for greater representation of fathers in paediatric hearing research [19]. A study examining fathers’ perceived reasons for their underrepresentation in child health research found that 248 of the 303 fathers surveyed had never been asked to participate [28]. This finding also highlights how it is necessary to explore how support needs differ for fathers and the secondary caregiver.

### 4.5. Varying Views on the Value of Parent Mentor Support

Some parents found parent mentor support to be one of the most valuable forms of help, whereas other parents found connecting with other families to be confronting. There are similarities between the attitudes expressed by parents in this study and those described by other parents interviewed in previous research who were also hesitant to access parent mentor support [19]. In the present study, one family thought that the timing of being offered parent mentor support immediately following the confirmed diagnosis was a factor in why they did not want to access it. This result has not previously been described and suggests that the timing is integral to how receptive parents may be to parent mentor support.

Additionally, an interesting finding was parents’ utilisation of social media, such as Facebook groups, to feel supported and connect with other families living similar experiences. Comparison of this finding with studies of children with special needs and those undergoing genetic testing confirms that both parental emotional and informational needs can be fulfilled by vetted online support platforms [29,30]. Social media can promote connection as well as practical tips on how to manage their child’s diagnosis [29,30]. In fact, online social support has shown to reduce feelings of isolation and even help parents come to terms with their child’s diagnosis as they feel more connected with others who understand what they are going through [29,30]. These ideas were reiterated by participants in this study. Interestingly, this informal form of parent mentor support was spoken about by those who were opposed to the formal parent mentor support on offer. As society becomes more connected through social media, this could be an area support services could utilise further to maximise the benefits of accessing families with lived experience of hearing loss. If a UNHS program does direct families to this kind of support, it is of utmost importance that families are directed to verified and good quality online support groups.

There were several strengths of this study. Parents were interviewed shortly after their newborn’s confirmed hearing loss diagnosis, specifically within three to six months post-diagnosis. This allowed for more accurate recall of their support needs along the diagnostic pathway. Offering telephone or Zoom interviews increased accessibility for regional families as well as allowing flexibility for interview scheduling. Importantly, this study included both regional Victorian families and fathers, allowing the sample to represent a more diverse range of experiences. All families whose newborns were diagnosed with bilateral moderate–profound sensorineural hearing loss within the timeframe of the study were invited to participate.

Limitations of the study include a short time frame for data collection and analysis, which in turn restricted the number of participants able to be interviewed. Additionally, the recruitment period was impacted by an extended Victorian lockdown due to COVID-19, which limited the ability for some parents to participate due to increased demands, such as home-schooling other children. As in other research, it was difficult to recruit fathers for an hour-long interview, especially if they had returned to full-time work. This study did not have funding available for interpreters, which prevented parents who did not speak English from participating. Despite the small sample size, data saturation was achieved.

This project has highlighted several areas where further research could contribute to clinical practice. One such area would be prioritizing the recruitment of both parents to understand how their support needs differ. This suggests that future research needs to include the recruitment of the other parent. This would allow a better understanding of the experience and support needs of both parents along the hearing diagnosis pathway, to ensure their broader needs are recognised. This might be particularly salient when the circumstances of caregiving or work might preclude one parent’s contact with health professionals. Specifically, the way to address the under-representation of fathers in research would be to directly contact the fathers rather than relying on indirect recruitment methods through the mother as done in this study. Perhaps utilising a different methodology such as a quantitative or qualitative questionnaires that they can complete in their own time would also increase participation rates in this cohort.

In addition, this study only explored the support needs of parents whose newborn had a congenital bilateral moderate to profound sensorineural hearing loss. Future research could explore whether parental support needs are different for parents of infants with different types of hearing loss, such as unilateral loss and mild loss, and for families who infants are referred to diagnostic audiology and are found to have normal hearing.

Due to the mixed response to parent mentor support, further exploration into the timing of this service would be valuable. These findings suggested that being offered this form of support in the early days was confronting and overwhelming, although parents do value having access to someone with lived experience. Another avenue of research that goes beyond the scope of this study would be to explore the parent experience of early intervention services as some parents alluded to areas they would like to provide feedback on.

### 4.6. Implications for Practice

This study has multiple implications for practice that have been divided into three areas: integrated support as a model for other UNHS programs, the delivery of information, and a referral pathway to therapeutic support.

#### 4.6.1. Integrated Support as a Model for Other UNHS Programs

These findings could provide a learning opportunity for other UNHS programs worldwide, especially for those programs that do not have an embedded support service. Based on the findings from this study, an embedded support service enables the informational and emotional needs of families to be met. Having a reliable allocated support worker who can provide guidance as well as a safe place to debrief parents’ concerns was shown to be important in helping parents adjust to their child’s hearing diagnosis. Access to a support worker also allows parents to not feel lost and overwhelmed during the busy diagnostic pathway. Additionally, the delivery of healthcare that aligns with the family-centred care framework can help ensure families feel supported and receive the best possible care for their child.

#### 4.6.2. The Delivery of Information

Findings from this study highlight the importance of modifying the way information is delivered from hearing screeners to parents. Our findings suggest that hearing screeners scripts could be reviewed to consider reframing the way information is delivered to parents after the newborn hearing screen. Moving forward, hearing screeners could be engaged in focus groups to understand their perspective about the parents’ emotional state and how to develop strategies to identify when parents would benefit from extra information. Involving both the healthcare provider and the family in designing and developing the best way to deliver information also aligns with fundamental principles of family-centred care.

#### 4.6.3. Referral Pathway to Therapeutic Support

Our findings suggest a referral pathway to access specialised psychologists or counsellors to support parents’ emotional needs could also be beneficial. Every parent will have different emotional needs, and some may not need extra psychological support. However, for those families where the journey was more complicated and the diagnosis was unexpected, it seems further mental health support may be necessary. The service could consider providing parents with a list of recommended providers and their contact numbers so parents feel supported to access a service. If parents are left to source psychological support themselves, this could be a barrier to accessing this form of help at a time when they are already overwhelmed. Alternatively, the VIHSP could consider integrating a counsellor or psychologist into the already-existing system to provide this support to parents who need it. A pilot program could be implemented to determine if parents would access this form of support and at what times this support is most beneficial. Additionally, further training for support workers to assess when supplementary therapeutic support for parents might be warranted could be helpful.

## 5. Conclusions

This study specifically explored parental support needs following their newborn’s diagnosis of bilateral moderate–profound sensorineural hearing loss. The main goal of this study was to understand the parent experience and their support needs from the newborn hearing screen until enrolment into early intervention services. The key findings from this study have direct implications for practice to help enrich the services VIHSP provides to ensure families receive the best standards of care. By identifying that most parents were satisfied with the support received, this study can provide a model for other UNHS services, especially those without an integrated support service. Moreover, the delivery of information following the newborn hearing screen and a referral pathway to therapeutic services are two additional areas where the service could be further enhanced. Importantly, the findings highlight that the current support on offer is meeting most of the support needs described by parents.

## Figures and Tables

**Table 1:** Participant characteristics.

Characteristic	Value
Infant (*n* = 10)	
Age in months at time of interview, median (range)	6 (5–11)
Male, *n* (%)	8 (80%)
Number of infants diagnosed with additional medical condition/s, *n* (%)	2 (20%)
Number of audiology appointments to reach final diagnosis, median (range)	1 (1–6)
Parent (*n* = 13)	
Age in years, median (range)	31 (25–37)
Mothers: Fathers, *n* (%)	10 (77%): 3 (23%)
Resided in metropolitan Melbourne, *n* (%)	11 (85%)
Spoke a language other than English at home, *n* (%)	4 (31%)

## Data Availability

Additional data are available from the corresponding author on request.
